# Ethnicity/Race and Age-Specific Variations of Serum AMH in Women—A Review

**DOI:** 10.3389/fendo.2020.593216

**Published:** 2021-02-09

**Authors:** Alexander M. Kotlyar, David B. Seifer

**Affiliations:** Section of Reproductive Endocrinology and Infertility, Department of Obstetrics, Gynecology, and Reproductive Sciences, Yale University, New Haven, CT, United States

**Keywords:** ovarian reserve, age, race, ethnicity, AMH, age-specific AMH levels

## Abstract

**Purpose of Review:**

In this review, we summarize ethnic/race- and age-related variation in AMH and discuss the underpinnings behind these differences.

**Recent findings:**

Anti-mullerian hormone (AMH) has become a widely used method of ovarian reserve testing over the last 15 years. Numerous studies have shown substantial ethnic/race and age-related differences. When compared to age-matched Caucasian women, AMH levels tend to be lower in black and Hispanic women. Chinese women tend to have significantly greater AMH levels prior to age 25 than Caucasian women. When considering subpopulations within ethnicities, at least one study noted lower AMH levels among Maya women compared to other Hispanic women. Age exhibits a positive trend with AMH up until at least 25 years of age with a consistent decline after 34 years of age extending to menopause.

**Summary:**

AMH levels are highly variable among ethnicities and race with higher age-matched levels typically seen in Caucasian women. Age does not exhibit a consistent linear relationship with AMH, but a consistent decline is seen starting in the third decade of life and proceeding to menopause.

## Introduction

Anti-Müllerian hormone (AMH) as a marker of ovarian reserve is an essential aspect of infertility testing. This hormone which is also known as Müllerian inhibitory substance/factor (MIS/MIF) was first discovered in 1947. Since 2002, the role of AMH/MIS has expanded from its influence on Müllerian ducts to a method of gauging a woman’s ovarian reserve (1).

AMH is part of the Transforming Growth Factor-beta (TGF-β) group of ovarian growth factor ligands. This family includes inhibins, bone morphogenic proteins (BMPs), activins, and growth and differentiation factors (GDFs). The 2750 bp gene for AMH is on the short arm of chromosome 19 which produces a 140kDa homodimer glycoprotein (2, 3). In-utero, AMH leads to the regression of the Müllerian ducts (4). In a male fetus, the SRY region of the Y-chromosome is expressed at approximately 8 weeks of gestation which then leads to AMH production in Sertoli cells. Once it has reached the Müllerian ducts, AMH causes the apoptosis of cells in these structures thereby leading to their regression. Due to the lack of the SRY region in female embryos, the Müllerian ducts continue to develop into the uterus, fallopian tubes, and upper 2/3 of the vagina (5). Nonetheless, AMH is produced within females and is exclusively made by the granulosa cells within pre-antral and antral follicles (6) and was discovered to be present in women’s pre-ovulatory follicles in 1993 (7).

Numerous unique properties have made serum AMH a mainstay of assessing a patient’s ovarian reserve. In particular, AMH as a measure of ovarian reserve is more reliable due to its reduced variation within each menstrual cycle and reduced interobserver variability compared to antral follicle count (AFC) and follicle stimulating hormone (FSH) levels (7–9). Additionally, AMH demonstrates minimal cycle-to-cycle variability in comparison to AFC and FSH levels (10, 11).

Given this greater consistency, AMH has become a widely used tool to assess ovarian reserve (8, 12). Furthermore, it has been studied as a tool to guide and assess the potential oocyte yield prior to ovulation induction for assisted reproductive technology (ART) cycles. The first study to demonstrate a reliable correlation between AMH and egg yield showed that AMH levels were 2.5 times higher in patients whose ART cycles yielded 11 or more oocytes compared to those that yielded 6 or less oocytes. This correlation between ART success and AMH was extended to live birth rates. A retrospective study looking at 1230 in-vitro fertilization–intracytoplasmic sperm injection (IVF-ICSI) cycle outcomes indicated that the likelihood of live-birth increased in a log-linear fashion for an of AMH 2.94 and greater (13). Numerous additional studies have confirmed the correlation between higher AMH levels and superior ART cycle outcomes (14–16).

Ethnicity/race and age can have a substantial impact on ovarian reserve and thus, oocyte yield during ART cycles (17–19). In this review, we intend to address how AMH may vary by ethnicity/race and varies according to age.

## Methods

A search was performed in Pubmed, The Cochrane Library, and Ovid-Medline. Phrases used in the search were suited for each individual database and included “AMH AND Caucasian quality,” “AMH AND African-American,” “AMH AND black,” “AMH AND asian,” “AMH AND race,” “AMH AND ethnicity,” “AMH AND age,” “AMH AND adolescents,” “AMH AND menopause,” “Müllerian inhibitory substance/factor” AND Caucasian,” “Müllerian inhibitory substance/factor AND African-American,” “Müllerian inhibitory substance/factor AND black,” “Müllerian inhibitory substance/factor AND Asian,” “Müllerian inhibitory substance/factor AND race,” “Müllerian inhibitory substance/factor AND ethnicity,” “Müllerian inhibitory substance/factor AND age,” “Müllerian inhibitory substance/factor AND adolescents,” and “Müllerian inhibitory substance/factor AND menopause. Our search period spanned from 1946-2020. 3352 articles were found. These articles were then assessed for relevance and quality. Only studies published in English were included. Thirty-three of these studies were included as part of this review. A manual review of the references in each of the cited sources was performed to ensure that any relevant resource was not excluded.

The primary outcome of this review was to determine if AMH levels were correlated at any age range and were associated with any ethnic/racial group. Articles were selected as relevant if they were: 1) prospective studies, retrospective studies or meta-analyses involving females who underwent AMH assessment between birth until menopause. Studies were excluded if they were 1) case reports, non-systematic reviews, abstracts, expert opinion articles, 2) did not include an analysis of patients that had an AMH drawn or only analyzed patients that has other markers of ovarian reserve such as AFC or basal FSH.

### Ethnicity/Racial Differences

As described in the following sections, ethnicity/race has been associated with substantial variations in AMH levels. Ethnicity/race has typically been determined *via* patient self - reporting. However, genetic markers of ethnicity/race, also known as ancestry informative markers (AIMs), have been seen as a more objective method of ascertaining a patient’s ethnicity. Olcha et al. looked at various ovarian reserve markers across various ethnicities based upon genetic ancestry *via* AIMs. This group showed that when controlling for age and body mass index (BMI), there is no variation in AMH based upon genetic markers of ethnicity (20). Determining ancestry *via* AIMs does have limitations which include the limited reference dataset of single-nucleotide polymorphisms that are used to identify genetic ethnicity (21). Consequentially, the use of AIMs may artificially contract the cohort of patients assigned to any one ethnicity. Therefore, we will assess the available literature on variations in AMH levels based upon patient-reported ethnicity.

#### Caucasian

Caucasian women are often the reference standard when assessing ethnic differences for a variety of parameters in infertility (22). Hence, we will first assess this ethnic/racial group. The first study looking at ethnic differences in AMH levels showed higher levels in Caucasian women compared to Black and Hispanic women after controlling for age, BMI, smoking, and HIV status (23). The second and one of the largest comparative assessments of ovarian reserve values among ethnicities was performed by Bliel et al. In this cross-sectional study they assessed AMH levels in 947 women, of which 277 were white, 237 were African-American, 220 were Latina, and 213 were Chinese. Compared to all other ethnic groups, average AMH levels were consistently greater in white women, until age 35 (24). Substantial research has looked to variations in AMH levels in Caucasian women according to the presence of polycystic ovary syndrome (PCOS). Moy et al. performed a retrospective analysis looking at factors that affect AMH among numerous racial/ethnic groups. While age did correlate negatively with AMH among all ethnic groups, the prevalence of polycystic ovary syndrome, smoking, and elevated BMI correlated negatively only in Caucasian women (25). Of note, no distinction was made between infertility status of the women in any of the aforementioned studies.

#### African-American/Black

Numerous studies have assessed AMH levels according to pre- or post-menopausal status. Based upon the study by Seifer et al., AMH was 25.2% lower in Black women compared to Caucasian women which was independent of age, BMI, smoking, and HIV status (23). In the study by Bleil et al., this group showed that their cohort of 237 Black and 213 Chinese women of younger and middle ages exhibited lower AMH levels compared to 227 white women. However, AMH levels in black women were higher than Latina and Chinese women of an older age (24). Marsh et al. looked at factors which lead to the variation in AMH levels in 1,654 African-American Women (AAW). Median AMH for AAW was 3.18 ng/ml and, in their age-adjusted model, they showed that BMI, use of hormonal contraceptives, and history of a thyroid disorder were negatively correlated with AMH levels. Furthermore, a history of abnormal bleeding during menses and oligomenorrhea was associated with higher AMH levels (26). Just as in Caucasian women, obesity, especially if patients were obese at 18 years of age had a significant negative correlation with AMH levels (27). This variation in AMH may be one contributing factor among several underlying the consistently lower live birth rates from assisted reproductive technologies observed in black women in the US (28). Looking at post-menopausal women, a cross-sectional study which included 671 women without a history of malignancy showed that race was not significantly associated with AMH levels (29). Furthermore, the study by Bleil et al. showed in a cohort of 947 women that AMH levels varied in a more consistent fashion across ethnicities among older women, especially when approaching the age of perimenopause (24). As with the studies in assessing AMH in Caucasian women, the studies assessing AMH levels in Black women did not distinguish between fertile and infertile populations.

#### Hispanic

As with black women, substantial differences have been noted in AMH levels with Hispanic women compared to other ethnic groups. Although the difference was not statistically significant, Seifer et al. showed a 24.6% lower AMH level in Hispanic women than in Caucasian women (22). Bleil et al. showed that, across all ages, AMH was lower in a cohort of 220 Hispanic women compared to the 227 Caucasian women included in the study (24). Even among Hispanic women, substantial variations in AMH levels exist. In one study looking at women of Maya heritage in Mexico, these women were over five times more likely to have undetectable AMH levels compared to non-Maya women (30). Further research into regional or ancestral differences, especially throughout the Caribbean, Central, and South America may yield additional nuances in ovarian reserve levels in this population.

#### Asian Populations

Examining populations throughout Asia, there are two distinct trends seen. In a Chinese study looking at over 6,700 women from birth to the post-menopausal age, the authors noted a peak in AMH at age 18 with a consistent decline up until age 50 (31). When compared to Caucasian women, Nelson et al. recently noted that AMH levels were typically higher in Chinese women up until 25 years of age and then AMH levels tended to be less than Caucasian women after age 25 (32).

This is not the case in the South Asian population. Bhide looked at AMH variations in 865 women at a single fertility clinic and showed that despite higher AMH levels seen in South Asian patients compared to Caucasians, this difference disappeared in their multivariable analysis (33). These results are consistent with data from ART cycles which showed no significant difference between 236 Indian women and 236 Spanish women as far as AFC. AMH was slightly lower among Spanish women, but this was likely a factor of their older age (34). While differences may be present between younger Chinese and Caucasian women, this disparity disappears in older women and especially within other populations in Asia.

#### Genetic and Environmental Underpinnings Behind Ethnic/Racial Differences

Several groups have looked relationships between AMH levels and genetic variations among different ethnicities. Schuh-Huerta et al. looked at genetic variants and AMH levels in 232 Caucasian women and 200 African-American women. They showed two nominal genetic variants in the Jumonji, AT-rich interactive domain 2 (JARID2) gene and AMH levels in both ethnic groups (35). A separate group looked at fragile-X mental retardation (FMR1) and found that AMH level was associated with the number of repeats in the FMR1 gene (p < 0.001) (36). This group correlated FMR1 variants with ethnicity and found substantial differences with Caucasians having the highest prevalence of abnormal alleles in this gene (37). BRCA1 has also been associated with lower AMH levels and BRCA1 is known to be more prevalent among non-Hispanic whites, and African-Americans (38, 39). Currently, it is unclear if any of these genetic factors lead to lower AMH levels primarily as a result of ovarian aging or rather as a result of altered AMH expression/secretion. Interestingly, at least one paper noted a paradoxical increase in AMH mRNA and protein in cumulus cells and follicular fluid as patient age increased (40).

Environmental factors may certainly also influence the ethnic variation of AMH. Numerous studies have shown a negative association between obesity, smoking and AMH levels (41–43). A multicenter study looking at the effect of BMI on the AMH levels of patients with PCOS and ovulatory controls, showed that BMI exhibited an inverse relationship with AMH regardless of patients age, race, smoking status and site in their regression analysis (44). This was seen in both cohorts. Plante et al. in their single-center cohort study looking at the effect of self-reported smoking history on AMH showed that current, but not past smokers have a 44% lower AMH level. Of note, they did not notice a dose-dependent response (45). Furthermore, these factors are known to be more prevalent in the African-American and Hispanic populations (46). Additional nutritional factors may include vitamin D deficiency which is more prevalent among women of color and may in part contribute to lower serum AMH levels in these patient populations (46, 47). However, additional mechanistic studies of these factors are needed to confirm these environmental/nutritional effects as direct drivers behind ethnic/racial disparities in AMH levels.

### Age-Related Differences

#### From Infancy to Adolescence

AMH exhibits a progressive trend in the early stages of life. A Danish study from 2010 in examined AMH levels in 926 healthy females from birth to adulthood. They noted that AMH was undetectable in 54% of cord blood samples (i.e. from infants). AMH then increased from birth to 3 months up to 15 pmol/liter (6.6 ng/ml). Then from 8-25 years, AMH levels remained stable with the average level being 19.9 pmol/liter (8.8 ng/ml) (48). This is consistent with a subsequent study which showed no difference in AMH levels in the American female population between 10–21 years of age (49).

#### Adulthood

Concerning AMH levels in older women, Lie Fong et al. (45) expanded on the work done by the Hagen et al. (45) by looking at AMH levels from birth to older adult ages. They noted that AMH climbed to its maximum by 15.8 years of age and then remained stable until 25 years of age at which point it started a progressive decline to menopause. With older women, AMH was negatively correlated with age (50). This correlation was modeled in a linear fashion; however both Lie Fong et al. and La Marca et al. showed that the relationship is better fitted with a polynomial function (50, 51). Overall, the relationship between AMH and age cannot be considered linear throughout all age ranges.

**Figure 1:**
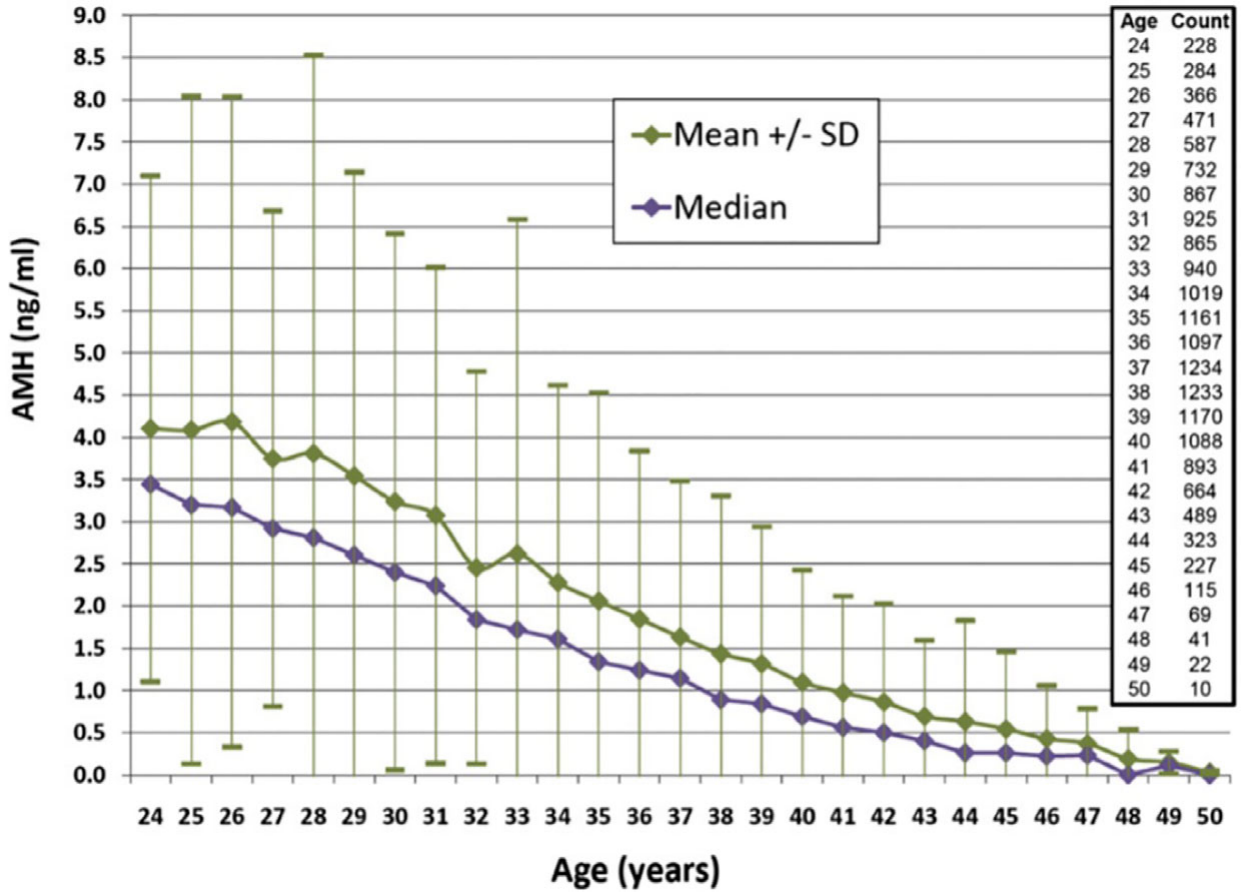
AMH levels according to age for women 24–50 years of age. Median values are shown with standard deviations. Reproduced with permission from Seifer et al. Age-specific serum anti-Mullerian hormone values for 17,120 women presenting to fertility centers within the United States. Fertil Steril. 2011; 95 (2): 747-50.

When considering ovarian reserve and fertility, it is essential to assess when the decline in AMH becomes more precipitous. Wiweko et al. (49) did a retrospective study that looked at the relationship between various markers of ovarian reserve including FSH, AFC, and AMH. Serum AMH was shown to decline after 34 years of age. Bozkurt et al. (50) also noted an age-related decline in AMH in both the fertile and infertile patients they studied. While they did not note an age at which the decline in AMH accelerated, patients 35 and older maintained a consistent age-related decline in AMH in both the fertile and infertile groups. Concerning the magnitude of this decline in the infertile population, the rate of decline of the median AMH was noted to decrease in a retrospective review of over 17,000 infertile women from 0.2 to 0.1 ng/ml per year after age 35 (52). Please see **Figure 1**. A subsequent prospective study comparing infertile women younger than 40 and fertile controls of the same age range noted an approximately 6% decrease in AMH per year (53). These findings supplement the findings in the infertile population from Seifer et al. (52). Thus, there are age-specific values that are clinically useful to keep in mind when using AMH in the infertile population to determine the status of a woman’s ovarian reserve. Of note, a retrospective study of healthy, reproductive age women showed that even at younger ages, concerningly low AMHs were noted. This suggests that even young women do have some risk for low ovarian reserve and that caution is needed when counseling patients on fertility planning (54).

### Clinical Significance

Our knowledge of the ethnicity- and age-related variation in AMH allows for giving proper context to patients. While patients of various ethnicities may exhibit lower levels of AMH, patients can be reassured that, the general trend of AMH with age appears to be consistent among ethnicities. Furthermore, the age-based variation is an effective tool to gauge possible oocyte yield for patients undergoing treatment with assisted reproductive technologies (55). The age-dependent decline in AMH can also assist in gauging response in women undergoing fertility preservation (55).

## Conclusions

AMH, as a mainstay of ovarian reserve testing, shows widespread ethnicity/race- and age-based variation. Variations according to ethnicity/race may be dependent on additional factors such as BMI, PCOS, socioeconomic status, environmental/nutritional factors such as vitamin D status among other aspects. However, the effect of age on AMH appears to be consistent among various ethnicities/races and thus, age-specific AMH values provide clinical context particularly in the infertile woman. Age is a strong common denominator that influences the reproductive life of women regardless of race or origin. Future research may shed light on how to mitigate the effects of ethnicity/race and age upon AMH as a reflection of ovarian reserve. This future work could potentially have a favorable impact on ART and fertility/family planning in general.

## Author Contributions

AK and DS contributed equally to the article search, analysis, review, and compilation of relevant articles for this review. All authors contributed to the article and approved the submitted version.

## Conflict of Interest

DS receives royalties from a licensing agreement between Beckman-Coulter and Rutger’s Medical School/MGH for the use of AMH in determining ovarian reserve.

The remaining author declares that the research was conducted in the absence of any commercial or financial relationships that could be construed as a potential conflict of interest.
